# Nuclear deformation by microtubule molecular motors

**DOI:** 10.1371/journal.pcbi.1012305

**Published:** 2025-05-08

**Authors:** Naruemon Rueangkham, Miguel Valle-Inclán Cabello, Franziska Lautenschläger, Rhoda J. Hawkins

**Affiliations:** 1 School of Mathematical and Physical Sciences, University of Sheffield, Sheffield, United Kingdom; 2 Department of Physics, KOSEN-KMITL, King Mongkut’s Institute of Technology Ladkrabang, Bangkok, Thailand; 3 Faculty of Natural Science, Saarland University, Saarbrücken, Germany; 4 Center for Biophysics, Saarland University, Saarbrücken, Germany; 5 African Institute for Mathematical Sciences, Accra, Ghana; Georg-August-Universitat Gottingen, GERMANY

## Abstract

We present a model to calculate the displacement and extension of deformable cellular cargo pulled by molecular motors stepping along cytoskeletal filaments. We consider the case of a single type of molecular motor and cytoskeletal filaments oriented in one dimension in opposite directions on either side of a cargo. We model a deformable cargo as a simple elastic spring. We simulate this tug-of-war simple exclusion process model using a Monte Carlo Gillespie algorithm and calculate the displacement and extension of the cargo for different configurations of motors and filaments. We apply our model to kinesin-1 motors on microtubules to investigate whether they are strong enough to translocate and deform the largest cellular cargo, the nucleus. We show that the extension caused by motors on a single microtubule saturates for larger numbers of motors but that the extension and displacement scales linearly with the number of microtubules. We also show how the binding and unbinding behaviors of molecular motors on microtubule filaments affect the nuclear deformation. Our modelling results correspond to experiments on cells treated with the drug kinesore, which is thought to increase rescue events resulting in more stable microtubules and more active kinesin-1 molecular motors bound to them. Both the experiments and our simulations result in nuclear deformation.

## Author summary

We developed a model to study how proteins called molecular motors transport cargo inside biological cells. These motors move along a network structure made of filaments, called microtubules, which are like highways. We focus on how these motors can stretch and move the cell’s largest cargo, the nucleus, which contains the genetic material. In our model, we treat the nucleus as a spring that can stretch when pulled. We’d like to know whether the molecular motors pulling force is strong enough to change the nucleus’s shape. How much will the nucleus stretch when the motors work in teams like a game of tug of war? How strong will they be if there are many microtubules in a bundle each with a team of motors (like multiple lanes on a highway)? Using computer simulations, we explore how different numbers of motors and microtubules affect the cargo. We found that with many motors on a single track, the stretching reaches a limit. However, having more tracks leads to greater stretching and movement. Our findings align with experiments of cells treated with a drug called kinesore. This drug stabilizes microtubule filaments and increases motor activity, leading to increased nucleus stretching.

## 1. Introduction

Vesicles, organelles and other intracellular cargoes are transported along cytoskeletal filaments in eukaryotic cells by molecular motor proteins such as kinesins and dyneins along microtubules [1, 2, 4, 6]. This active, directed intracellular transport is important when diffusion is insufficient due to time or space requirements. Sometimes, different motor types can be simultaneously bound to the same cargo and move in opposite directions following the polarity of microtubules. For example, kinesin-1 is directed towards the plus-end of microtubules and dynein is directed towards the minus-end of microtubules. This leads to bidirectional transport described as a tug-of-war model between the two species of motors [7–9]. Competition between two oppositely directed types of motor results in intracellular cargoes, such as mitochondria and mRNA complexes, being transported in the dominant direction. If forces from opposite teams of motors pulling the cargo are balanced, a paused state occurs. Interestingly, Gennerich *et al* [8, 9] found that paused cargo (mitochondria) were deformed in their experiments. Fernández Casafuz *et al* [10] classified mitochondria deformation fluctuations in living cells. Microtubules and motors are thought to be associated with mechanical forces on organelles such as mitochondria [10, 11]. However, mechanisms underlying vesicle elongation and cargo deformation have not yet been widely investigated.

Over the past few decades, behaviours of molecular motors in transporting a cargo have been extensively studied using theoretical models [4, 7, 12–18]. Most previous models have not explicitly considered the properties of the cargo nor how molecular motors impact the cargo itself, in particular the possibility that the cargo could be deformed. Intracellular cargo has generally been assumed to be a rigid solid that cannot change shape. One notable exception is a recent one-dimensional model for mitochondria transport and deformation [19]. Here in our work we relax the rigid cargo assumption in order to explain experiments in which cargo deformation is observed [8–10].

The largest organelle in eukaryotic cells is their nucleus. It is well known that cell nuclei displace and deform [20–24] but the mechanisms by which cells achieve this are not known. Nuclear displacement and deformation are important because the nucleus needs to change shape to move through constrictions in metastasis and invasion of cancer cells. Therefore, understanding mechanical and biophysical mechanisms underlying nuclear movement and deformation could potentially lead to improved treatment of cancer [25, 26]. We posed the question of whether molecular motors stepping along cytoskeletal filaments are strong enough to displace and deform the nucleus. This question is non-trivial since the nucleus is much larger than organelles such as mitochondria and therefore any displacement would have to overcome a much larger drag force. In terms of deformation the nucleus has an elastic component which is known to be around ten times stiffer than the rest of the cell [27] and is therefore much harder to deform. Are molecular motors strong enough to deform the nucleus? If so, how many molecular motors are needed to produce sufficient forces? In this article we answer these questions using computational simulations and experimental data.

In recent years much experimental work has been done on nuclear mechanics. An elegant technique to measure nuclear mechanics is by aspiration into a micropipette [28, 29]. This shows that the nucleus is deformable and suggests that the nucleus behaves like a viscoelastic solid [28, 30]. Stephens *et al*. [25] used a micropipette technique to measure an associated stiffness parameter describing nuclear elasticity. They attached a micropipette at one end of an isolated nucleus and at the other end attached a force reporting pipette. They reported the nuclear spring constant 0.52nN/μm from calculating the slope of the force-extension plot. A micropipette tip can even be used to apply a direct force probe into nuclei in cultured, living, adherent cells [31]. Neelam *et al* [31] sealed a micropipette tip to the nuclear surface and translated the pipette away from the nucleus, measuring the force required to do so. They find that to significantly displace and deform a nucleus a minimum pulling force of a few nanonewtons (2−3nN) is required, which is far greater than intracellular motor pulling forces [31]. Note that the stall force for a single kinesin-1 motor protein is 1−10pN [17] and therefore cannot significantly deform a nucleus. In this article we consider whether molecular motors working together in teams can produce sufficient force to significantly deform a nucleus.

There are various competing hypotheses for generating forces acting on nucleus[20, 32]. Candidates for force generation include actomyosin contractile forces that push and/or pull the nucleus, polymerisation of cytoskeletal filaments, extracellular forces applied to adhesion receptors and transmitted through the cytoskeleton and microtubule molecular motors (dynein and kinesin) [20, 30, 31]. Generally, nuclear movement will occur when there is a net differential in mechanical forces across the nucleus, while nuclear deformation will occur when the total extensile, compressive or shearing mechanical forces overcome the mechanical resistance of the nuclear material[30]. In stationary cells, nuclear shape and position are maintained in homeostasis in which the forces are constantly generated and balanced on the nucleus [31]. In this work we investigate the influence of microtubule motor pulling forces on the nucleus. It is known that the nucleus is connected to microtubules via kinesin and dynein motor proteins binding to KASH proteins [33]. Molecular motor proteins such as kinesin and dynein can exert active forces on the nucleus affecting its position within a cell[34–37]. In this article, we focus on kinesin motors. Some kinesin motors can perform a large number of steps before detaching and are thus processive motors [38–40]. They move towards the plus ends of microtubules (anterograde direction) [41, 42] with a strong preference for forward stepping over backward stepping. This means their direction is towards the cell periphery which is the plus end of microtubules’ polarity [38, 41].

In the work we present here, we have developed the tug of war model [7] of opposing teams of motors to study the effect of motors’ stepping behaviours on cargo deformation as well as cargo displacement. Our model focuses on kinesin-1, plus end-directed microtubule motors, moving on oppositely directed bundles of microtubules. This situation is similar to that of oppositely directed molecular motors on the same or parallel microtubules. We first assume the motors can be modelled as perfectly processive motors which never unbind from the microtubules [12, 18]. Then we extend the model to allow binding and unbinding to reflect the non-processive nature of kinesin-1. Note that in the biology literature “processive” means performs multiple steps before detatching opposed to “non-processive” perfoming only one step before detaching. However, in modelling there is a qualitative difference between never detaching and sometimes detaching. In contrast, detaching after only one step or multiple is a quantitative difference. In this paper we refer to “perfectly processive” meaning the idealised model of never detaching and “partially processive” to mean detaching with a given rate. Therefore our “partially processive” category covers the biological cases of “processive” (detaching after multiple steps with a low off rate) and “non-processive” (detaching after one step with a high off rate). Our results reveal the ability of molecular motor stepping to deform as well as displace cargo. We specifically consider the case of nuclear deformation.

## 2. Methods

**Fig 1:**
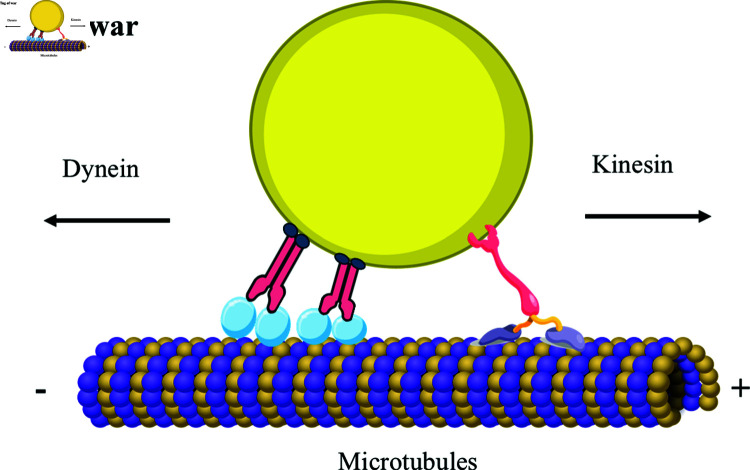
Schematic of tug-of-war between oppositely directed molecular motors. A single microtubule filament is depicted as a one dimensional lattice with its distinct polarity. Molecular motors (small circles) attached to a cargo (large yellow ellipse) exert force on it by stepping along the microtubule filament from one site to the next. Plus-end-directed kinesin-1 and minus end-directed dynein on the same microtubule pull in opposite directions. This figure was created using icons from the Reactome Icon Library (https://reactome.org/icon-lib) and SciDraw (https://scidraw.io), available under a Creative Commons Attribution 4.0 International (CC BY 4.0) license.

The tug of war model [7, 43] is often used to describe teams of opposing motors. A common example is plus-end-directed kinesins and minus end-directed dyneins on a microtubule, as shown in Fig 1.

**Fig 2:**
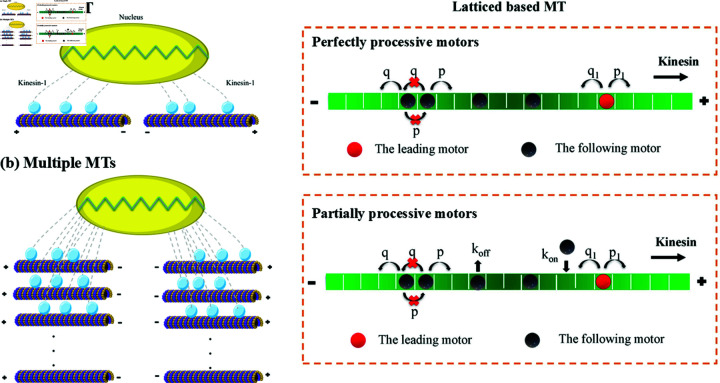
Schematic of tug-of-war between two opposite teams of the same type of molecular motors (kinesin-1, in blue) along (a) one and (b) multiple microtubules. The microtubules on the left are polarised in the opposite direction to those on the right. In the simulation, the microtubules are modelled as a one dimensional lattice on which kinesin-1s are either perfectly processive (never unbind off the microtubules) or partially processive motors (can unbind off the microtubules after one or multiple steps). Kinesin-1 motors on microtubules are connected to the cargo and attempt steps preferentially towards the microtubule plus-end, so the forward stepping rate, *p*_1_ and *p*, is much larger than backward stepping rate, *q*_1_ and *q*, for the leading motor and the following motors, respectively. The motors are not allowed to move to an occupied site thus obeying a simple exclusion process. For partially processive motors, they can bind on and unbind off the microtubules with a rate of $k_{\text{on}}$ and $k_{\text{off}}$, respectively. The cargo (large yellow ellipse) is allowed to deform elastically using a model of a simple elastic spring with spring constant, k=0.52nN/μm chosen for a cell nucleus from measurements by Stephens *et al* [25]. This figure was created using icons from the Reactome Icon Library (https://reactome.org/icon-lib), available under a Creative Commons Attribution 4.0 International (CC BY 4.0) license.

In biological cells microtubules are not always found as single filaments but are often arranged in bundles, e.g. in axons. Bundles of parallel microtubules can be modelled as a single coarse grained track [12, 44, 45] or by considering multiple lanes with the possibility of motors changing lanes [46, 47]. Here we consider cases of microtubules that are in opposite directions such that motors of the same type can act in opposing directions if they are bound to two opposing bundles of microtubules. We base our model on the tug of war paradigm, however we allow teams of motors comprised of the same type of molecular motors bound to bundles of microtubules in opposide directions. This is a bit different from the usual tug-of-war scenario composing of two types of differently directed molecular motors along the same microtubule. We model molecular motors that attach on either one or multiple microtubules on opposite sides of the cargo. An example of kinesin-1 motors bound on microtubules that are anti-parallel to those on the opposing side of the cargo is illustrated in Fig 2a and 2b.

The main novelty of our work is the study of cargo deformation. We assume the simplest possible model for a deformable cargo as a spring representing the response of a purely elastic material. This simple model is valid for any cargo for which an elastic spring model is reasonable. We are particularly interested in the largest possible subcellular cargo, the nucleus.

### 2.1. Model

Athough the model is general, we specifically consider the nucleus and the molecular motor kinesin-1, which moves along microtubules towards their plus end. During interphase in animal cells this usually results in the motors moving from the cell centre towards the cell edge due to the geometrical arrangement of the microtubules with their plus ends radiating outwards from the centrosome located near the nucleus [37]. Our choice of microtubule direction is inspired by this geometry but for simplicity we model filaments that are aligned parallel to each other and antiparallel to the ones on the opposite side of the nucleus, as shown in Fig 2.

To determine how motor pulling forces can move and/or deform the cargo, we assume that the motors are strongly bound to the cargo such that the shape and position of the cargo are changed following motors’ stepping. We do not allow motors to detach from the cargo during the course of our simulations. We assume that the motors performing steps on the microtubules generate force to pull the nucleus resulting in its deformation and movement. The force generated by motor stepping on microtubules is transmitted to the nuclear envelope at the right and left edges (as depicted in Fig 2). If the motors generate a net differential force between the right and left ends of nucleus, this will result in nuclear displacement and the sum of motor forces across the nucleus will result in nuclear deformation. The cargo either extends or displaces or both until motors reach their stall force and stop moving.

We simulate the motors as biased random walkers on a one dimensional lattice by using a Gillespie algorithm [48] as described in Sect 2.3. Motors move forwards with rate *p* and backwards with rate *q*. We implement a simple exclusion process [49] such that each lattice site can be occupied by a maximum of one motor, i.e. any attempted moves to an already occupied site are prevented from occurring. This is analogous to a short range hard core repulsion between motors at neighbouring sites. We also use the leading motor model [12] in which we assume the leading motor feels all the force and any additional motors following in the steps of the leading motor on the same microtubule do not feel any of the force. For simplicity we initially assume that kinesin-1 are perfectly processive without any detachment from microtubules during the simulation time. We use parameter values for kinesin-1 of forward rate $p=100s^{-1}$ and backward rate $q=10s^{-1}$ [12, 17, 50]. The leading motor’s rates depend exponentially on the force they experience, $p_{1}=pe^{-f\delta }$ and $q_{1}=qe^{f(1-\delta )}$, where *f* is dimensionless force and δ is a dimensionless fraction. The load force is given by $F=fk_{B}T/\text{d}x$ where dx is the motor step size. The fraction δ determines how much the forward versus backward stepping rates are affected by the load force. Due to simple exclusion process, the motors cannot overtake each other. Following the work of [12, 49] and our previous work [18], the velocity of *N* perfectly processive motors moving in one lane under dimensionless force *f* is given by

$$V_{N}=p\frac{\left(1-e^{f}(q/p{)}^{N})(1-q/p\right)}{e^{f\delta }(1-q/p)+e^{f}(q/p-(q/p{)}^{N})}.$$ (1)

For the extension of motor behavior to include detachment ($k_{\text{off}}$) and attachment ($k_{\text{on}}$) on microtubules with a limited number of binding sites *M*, the average velocity of *N* partially processive motors [18] is

$$V_{N}=\sum_{n=1}^{N}\frac{N!}{n!(N-n)!}\frac{M!}{(M-n)!}\frac{P_{0}}{1-P_{0}}{\left(\frac{k_{\text{on}}^{s}}{k_{\text{off}}}\right)}^{n}\left(\frac{p(1-e^{f}(\frac{q}{p}{)}^{n})(1-\frac{q}{p})}{e^{f\delta }(1-\frac{q}{p})+e^{f}(\frac{q}{p}-(\frac{q}{p}{)}^{n})}\right).$$ (2)

where $k_{on}^{s}$ is the binding rate of a single motor per binding site ($k_{on}^{s}=\frac{k_{on}}{M}$).

Previous models [4, 7, 12–17] including our own [18] have not considered the possibility that the cargo could be deformed. In this work, we focus on the study of cargo deformation in addition to displacement. We assume the simplest possible model for a deformable cargo as a linear elastic (Hookean) spring. As such our model can be applied to any cargo with mechanical properties consistent with that of a linear elastic spring. To apply our model to the nucleus we assume it acts as a Hookean spring. There is experimental support that the nucleus behaves as an elastic spring on the timescales of interest [25, 26, 51, 52].

In Fig 2a and 2b, kinesin-1 motors are simultaneously bound to the right and left ends of a spring (modelling the nucleus) and to microtubules in opposite direction on each side (plus ends outwards). The nucleus is pulled in opposite directions by the motors on each side as the motors step towards the plus ends of the microtubules. If the force generated by the motors is large enough, it can deform and/or displace the nucleus. The force generated by the motors is given by the effective spring constant of the nucleus and the distance the motors step along the microtubules. We calculate the stepping distance as the movement of the forwardmost leading motors of the right and left teams. The force on the spring modelling the nucleus is then $f_{\text{spring}}=kdx_{\text{extension}}$ where *k* is nuclear spring constant, which we take as $k=0.52nN\;\mu {\text{m}}^{-1}$ [25] and $dx_{\text{extension}}$ is the extension of the nucleus. The spring extension is given by $dx_{\text{extension}}=dx_{r}$  +  *dx*_*l*_ where *dx*_*r*_ and *dx*_*l*_ are the distances moved right by the forwardmost leading motor of right team and left by the forwardmost leading motor of the left team, respectively. The displacement of nucleus is determined by the difference between the distances moved by the right and left motors, $dx_{\text{displacement}}=dx_{r}$ − *dx*_*l*_. The sign of $dx_{\text{displacement}}$ shows the net direction of movement (positive towards the right). The nucleus will stop deforming and displacing when the motors of both teams stop moving i.e. the point when the motor velocity vanishes. For a team of motors on a single microtubule this is given by setting Eqs 1 and 2 to zero for perfectly processive motors and partially processive motors, respectively. Note that the initial size of the cargo in this model is arbitrary. The extension is unaffected by the initial size of the cargo. However, in a more realistic model, the displacement would be affected by the size of the cargo via the drag force. Such a model would require coupling the displacement velocity of the cargo to the force and is beyond the scope of this study. Our model is focused on the force due to cargo deformation and calculates how much our model nucleus is deformed and displaced by the force generated by molecular motors.

### 2.2. Simulation procedures

#### 2.2.1. Perfectly processive motors.

**Fig 3:**
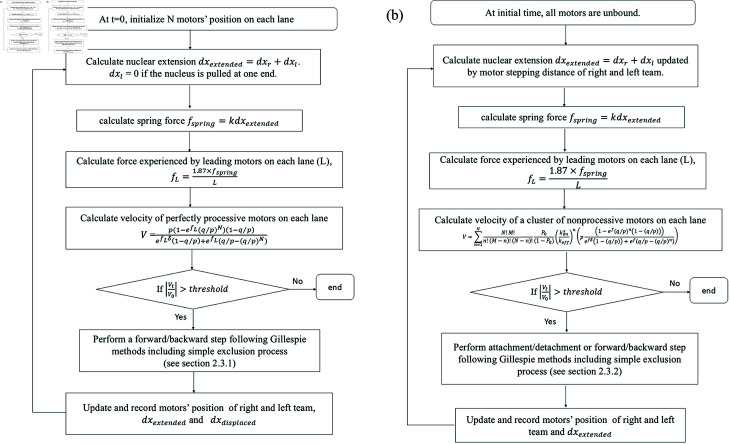
(a) Flow chart of our simulation of cargo extension and displacement pulled by perfectly processive motor clusters on right and left sides. (b) Flow chart of our simulation of cargo extension and displacement pulled by partially processive motor clusters on right and left sides.

To study the extension and displacement of a nucleus pulled at both ends by molecular motors connected to microtubules we simulate our model by performing the steps shown in the schematic in Fig 3a and described in more detail in the following.

1. Initialize motors’ positions randomly on each lane within a determined set of lattice sites.
2. Calculate the extension, $dx_{\text{extended}}=dx_{r}+dx_{l}$, and displacement, $dx_{\text{displacement}}=dx_{r}-dx_{l}$, of the nucleus. Initially, at time *t* = 0, $dx_{\text{extended}}=0$. Note that if the nucleus is pulled by only at the right end, then *dx*_*l*_ = 0.
3. Calculate the spring force, $f_{\text{spring}}=kdx_{\text{extended}}$ where $k=0.52nN\;\mu {\text{m}}^{-1}$ is nuclear spring constant [25] and $dx_{\text{extended}}$ is the extension of nucleus.
4. Calculate the dimensionless force experienced by the leading motor on each microtubule, $f_{L}=\frac{f_{\text{spring}}\text{d}x}{Lk_{B}T}$ where $f_{\text{spring}}$ is spring force, *L* is the number of microtubules (number of lanes) at the relevant side (*L*_*r*_ right/ *L*_*l*_ left), dx=8nm is the step size of kinesin-1, *k*_*B*_ is Boltzmann’s constant and T=310K is body temperature, so $f_{L}=\frac{1.87\times f_{\text{spring}}}{L}$. Note that *L*_*r*_ = *L*_*l*_ for a balanced pulling team, but *L*_*r*_ and *L*_*l*_ can be different for an unbalanced pulling team.
5. Calculate the velocity of the group of *N* motors on each lane as given by Eq 1 by substituting *f* = *f*_*L*_. If the normalized velocity, $\left|\frac{V_{N}(t)}{V_{N}(0)}\right|$, at time *t* of a motor cluster on each lane is less than a determined threshold, then the simulation stops. The force $f_{\text{spring}}(t)$ at this occurs is the stall force.
6. If $\left|\frac{V_{N}(t)}{V_{N}(0)}\right|$ is larger than the threshold then allow one of the motors to perform a step as described in detail in Sect 2.3.1.

#### 2.2.2. Partially processive motors.

We now construct simulation models to study the nuclear extension pulled by both teams of partially processive molecular motors on the left and the right. In this model, motors not only perform stepping, but are also able to bind and unbind from filaments. The simulation procedures are shown in the schematic in Fig 3b and described in the following steps.

1. At the initial time, all motors are unbound.
2. Calculate the nuclear extension $dx_{\text{extended}}=dx_{r}+dx_{l}$ from the stepping of the right and left teams. *dx*_*r*_ and *dx*_*l*_ are calculated by the distance between the forwardmost leading motor’s position and the first bound motor’s initial position for each side respectively. Note that at time *t* = 0,*dx*_*r*_ = 0 and *dx*_*l*_ = 0.
3. Calculate the spring force, $f_{spring}=kdx_{\text{extended}}$ where $k=0.52nN\;\mu {\text{m}}^{-1}$.
4. Calculate the dimensionless force experienced by the leading motor on each lane, $f_{L}=\frac{f_{\text{spring}}\text{d}x}{Lk_{B}T}$ where $f_{\text{spring}}$ is spring force, *L* is the number of microtubules (number of lanes) at the relevant side (*L*_*r*_ right/ *L*_*l*_ left), dx=8nm is the step size of kinesin-1, *k*_*B*_ is Boltzmann’s constant and T=310K is body temperature, so $f_{L}=\frac{1.87\times f_{\text{spring}}}{L}$.
5. Calculate the velocity of the group of *n* bound motors out of maximum *N* motors within a limited number of binding sites, *M*, on each lane as given by Eq 2 by substituting *f* = *f*_*L*_.
6. Calculate the normalized velocity, $\left|\frac{V_{N}(t)}{V_{N}(0)}\right|$, at time *t* of a motor cluster on each lane for the right and the left. If the normalized velocity of a motor team on lanes from either right or left side is less than a determined threshold, then the position of that team stays the same. If the normalized velocity of both teams is less than the threshold, then the simulation stops. The force $f_{\text{spring}}(t)$ at which this occurs is the stall force.
7. If $\left|\frac{V_{N}(t)}{V_{N}(0)}\right|$ is larger than the threshold then allow one of events to happen as described in detail in Sect 2.3.2.

### 2.3. Gillespie algorithm

To simulate our model of molecular motors’ stepping along filaments, we use a Gillespie algorithm, originally developed for chemical reaction systems [48, 53, 54]. The Gillespie algorithm is a method in which the time until the next event dt is drawn from an exponential distribution $\exp\left(-\alpha_{0}dt\right)$ with a rate parameter, $\alpha_{0}$, given by the sum of the rates of all possible events from the current state. Only one event happens at each time step and the duration of each time step is changed every iteration because it is drawn from the distribution according to a random number generated. The time step is calculated as $dt=\frac{1}{\alpha_{0}}\ln\frac{1}{r_{1}}$ where *r*_1_ is a random number drawn from a uniform distribution (0,1). Which event happens in that time step is determined by a second random number, *r*_2_, drawn from the uniform distribution.

#### 2.3.1. Perfectly processive motors.

In our perfectly processive model, motors step to neighboring sites with the forward and backward rates of $p_{1r},q_{1r}$ and $p_{1l},q_{1l}$ for the leading motor on the right and left respectively. The following motors step with rates of *p*_*r*_, *q*_*r*_ and *p*_*l*_, *q*_*l*_ respectively. Therefore, there are eight different rates for possible events, however, due to the simple exclusion process, not all these events will be possible. The algorithm proceeds according to the following steps:

1. Generate two random numbers from uniformly distributions (0,1): *r*_1_ to compute the length of the time step and *r*_2_ to choose which event happens.
2. Compute $\alpha_{0}$ for all the events,
  $$\alpha_{0}=a_{1}p_{1r}+a_{2}q_{1r}+a_{3}p_{r}+a_{4}q_{r}+a_{5}p_{1l}+a_{6}q_{1l}+a_{7}p_{l}+a_{8}q_{l}$$ (3)
  where *a*_1,...,8_ is the number of events allowed by the simple exclusion process for each motor type. For example, *a*_1_ is the number of leading motors (one on each lane) on the right side that have an empty site in front of them so are allowed to move forward. This number is then multiplied by the relevant rate, *p*_1*r*_ for the *a*_1_ term.
3. Compute the time when the next event takes place as t+dt where $dt=\frac{1}{\alpha_{0}}\ln\frac{1}{r_{1}}$
4. Compute which event happens in the current time step according to
  $$\text{if}\;r_{2}\leq \frac{a_{1}p_{1r}}{\alpha_{0}}\;\text{a right leading motors moves forward }x_{1r}=x_{1r}+1$$
  $$\text{if}\;\frac{a_{1}p_{1r}}{\alpha_{0}}\leq r_{2}\leq \frac{a_{1}p_{1r}+a_{2}q_{1r}}{\alpha_{0}}\;\text{a right leading motor moves backward }x_{1r}=x_{1r}-1$$
  and equivalently for all eight types of event. Note that in the case of a cargo being pulled at one end only, there will be only four types of event with the events associated with the other side vanishing.

#### 2.3.2. Partially processive motors.

In our partially processive model, we extend molecular motor behavior to not only stepping along microtubules, but also binding on and unbinding off the microtubules with a rate of *k*_*on*_ and *k*_*off*_, respectively. As for perfectly processive motors, the stepping is performed as described in Sect 2.3.1. Adding the binding and unbinding, there are now twelve possible events. The $\alpha_{0}$ for all the events of step 2 in Sect 2.3.1 changes as follows,

$$\alpha_{0}=a_{1r}k_{on}^{s}+a_{2r}k_{off}+a_{3}p_{1r}+a_{4}q_{1r}+a_{5}p_{r}+a_{6}q_{r}\;+a_{7l}k_{on}^{s}+a_{8l}k_{off}+a_{9}p_{1l}+a_{10}q_{1l}+a_{11}p_{l}+a_{12}q_{l}$$ (4)

where *a*_1*r*_ and *a*_2*r*_ are the number of events of the right motor team allowed to bind and unbind from filaments, respectively. *a*_7*l*_ and *a*_8*l*_ are the number of events of left motor team allowed to bind and unbind from filaments, respectively. $a_{3},a_{4},a_{5},a_{6}$ and $a_{9},a_{10},a_{11},a_{12}$ are the number of events allowed by the simple exclusion process for the right team and left team, respectively. For example, *a*_3_ is the number of leading motors (one on each lane) on the right side that have an empty site in front of them so are allowed to move forward. This number is then multiplied by the relevant rate, *p*_1*r*_ for the term *a*_3_.

In binding events, *a*_1*r*_ and *a*_7*l*_ are the total binding rates of all filaments of the right (*L*_*r*_) and left teams (*L*_*l*_) in which each of them can have a maximum *N* partially processive motors bound on a limited number of binding sites *M* along the filament. The total binding rate of each team is (NL − $\sum_{i=1}^{L}n_{i})(\frac{\sum_{i=1}^{L}M_{i}-\sum_{i=1}^{L}n_{i}}{\sum_{i=1}^{L}M_{i}})k_{on}$ where *i* represents an individual filament, *n* is the number of bound motors on the filament and *M* is the number of binding sites on the filament. If the binding event is chosen in a time step, then an unbound motor will be bound at an unoccupied site within the limited number of binding sites, *M*, on a lane. We set *M* = 20 if no bound motors are on the lane. However, once motors are bound *M* will change during the simulation due to (un)binding and stepping. We allow *M* to change following the leading motor and last motor’s positions at each step. We also add one more site available in front of the leading motor and two more available sites (one in front and one behind) for following motors. For the following motors, we allow them to swap position when rebinding. The motor sequence is always preserved during stepping following simple exclusion process but not during (un)binding.

In an unbinding event, *a*_2*r*_ and *a*_8*l*_ are the total unbinding rates off all filaments on the right (*L*_*r*_) and left (*L*_*l*_) sides. The total unbinding rate of each team is $(\sum_{i=1}^{L}n_{i})k_{\text{off}}$ where *n* is the number of bound motors on lane *i*.

### 2.4. Experimental methods

We performed experimental techniques following Gaudenz Danuser, *et al*. [55]. Live cell imaging was performed with a Nikon Eclipse Ti epifluorescent microscope using a 20x magnification objective. Prior to image acquisition, the temperature of the microscope incubation chamber was set to $37^{\circ }C$ and ${\text{CO}}_{2}$ levels were kept at 5 %. For experiments in cells, hTERT immortalised human Retinal Pigment Epithelial cells (RPE-1), in which vimentin and alpha-tubulin had been labelled with the fluorescent proteins mEmerald and mTagRFPt respectively, were used. These RPE-1 cells were kindly donated by the group of G. Danuser and cultured in T25 cell culture flasks (Thermo Scientific) with DMEM medium (GIBCO) supplemented with 10 % FBS, 1X penicillin/streptomycin (GIBCO) and 1X GlutaMAX (GIBCO).

For each condition, 30000 RPE-1 cells were resuspended in 2 ml of culture medium, pipetted onto microscope dishes and incubated for 5 hours at $37^{\circ }$C , 5% ${\text{CO}}_{2}$. Once the cells had adhered to the surface, the medium was carefully aspirated and cells were treated with 50μM Kinesore (SML2361, Sigma Aldrich) diluted in Ringer’s buffer 2 (MilliQ with 150mM NaCl, 2mM${\text{CaCl}}_{2}$, 10mM HEPES, 5mM KCl and 11mM glucose, pH: 7.4). Hoechst stain (H3570, Invitrogen) was added to the solution at a concentration of 200 ng/ml to visualise cell nuclei. Cells were incubated with the compound solution for 30 minutes, after which the solution was removed, and cells were washed twice with fresh Ringer’s buffer to remove any remaining residue from the treatment. We checked for viability using a Luna Dual Fluorescence Cell Counter and found no difference between kinesore treated and control cells viability.

## 3. Results and discussion

We performed Monte Carlo simulations using a Gillespie algorithm as detailed in Sect 2.3 to study the deformation and displacement of a cargo pulled by molecular motors stepping along cytoskeletal filaments. We assume the simplest possible model for a deformable cargo as a Hookean spring such that the cargo extension is linearly proportional to the force across the cargo. We focus on an example of the nucleus being pulled by kinesin-1 motors stepping along microtubules. For simplicity we take *N* perfectly processive kinesin-1 motors on each microtubule, which remain bound for the duration of the simulation. We simplify the geometry of a microtubule aster to that of parallel microtubules on the right antiparallel to parallel microtubules on the left as depicted in Fig 2. Furthermore, we also extended the complexity of the model by including the binding and unbinding behaviors of molecular motors on microtubule filaments and show the results in Sect 3.3.

**Fig 4:**
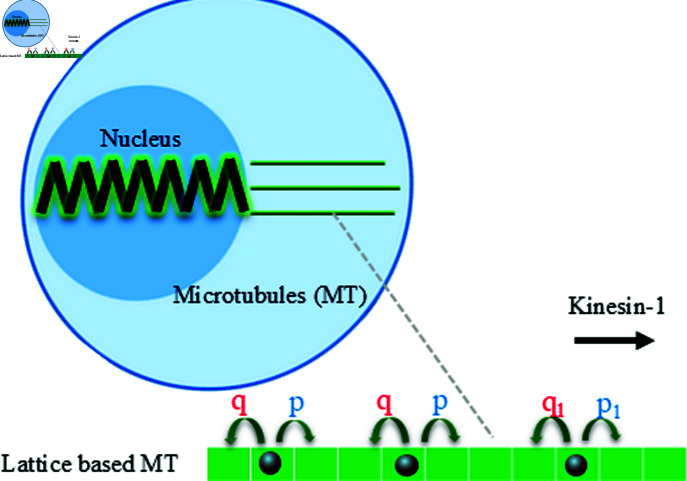
Diagram of a deformable cargo connected to three filaments (lanes, *L* = 3) at one end on which molecular motors move with forward (p) and backward (q) rates. The parameters, for kinesin-1, are $p=100s^{-1}$, $q=10s^{-1}$ and *d* = 0.5 [12, 17, 50] and the cargo spring constant is taken, for a nucleus, as $k=0.52nN\mu {\text{m}}^{-1}$ [25]. This figure was created using icons from the Reactome Icon Library (https://reactome.org/icon-lib), available under a Creative Commons Attribution 4.0 International (CC BY 4.0) license.

We begin with presenting the results of a model of a cargo pulled just at one end as illustrated in Fig 4 and then one pulled at both ends as illustrated in Fig 2.

### 3.1. Cargo pulled at one end

We initially consider a simple model of a cargo connected to a team of motors on a single or multiple lanes on the right as shown in Fig 4. In this case, the molecular motors pull in one direction toward the plus ends of the microtubules and the other end of the cargo is fixed. *N* kinesin-1 motors are bound to each microtubule. The force pulling and deforming the cargo is generated by steps made by the leading motor for a single microtubule and the forward most leading motor for multiple mircrotubules.

We investigate the effect of the number of motors, *N*, and the number of lanes, *L*, on the extension of the cargo. The amount the cargo is extended is given by the number of steps taken by the (forward most) leading motor. As the motors step further, the cargo is stretched more, which increases the force experienced by the leading motor, which consequently slows the motor stepping speed. Eventually the motor speed reaches zero and the motors stop. For practical reasons in our simulations, we choose a threshold velocity below which we assume the motors have stopped. In simulation tests we found results are dependent on the choice of threshold. We chose the threshold $|\frac{V_{N}(t)}{V_{N}(0)}|=10^{-5}$ because it provides the same accuracy as experimental results *in vitro* [17] and *in cellulo* [56].

**Fig 5:**
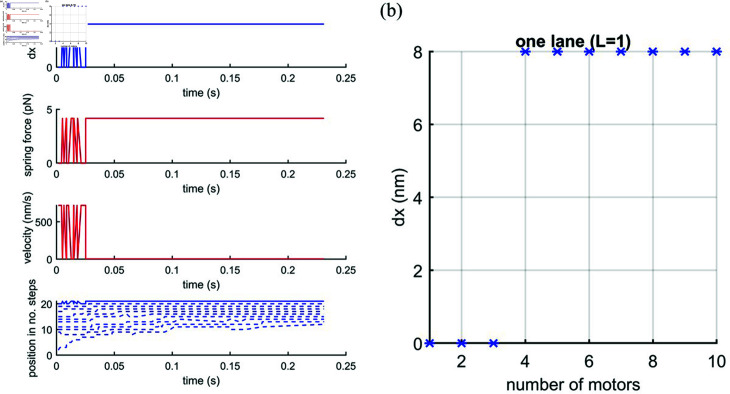
(a) An example simulation time course of cargo extension (dx), force (*f* = *kdx*), velocity and trajectories of motors (Blue solid line for the leading motor and dashed blue line for the following motors) of *N* = 10 motors stepping along one lane (*L* = 1). The spring constant is taken, for a nucleus, as $k=0.52nN\mu {\text{m}}^{-1}$ [25]. (b) Cargo extension (*dx*) against number of motors *N* on one lane (*L* = 1).

The first case we document is that of a cargo connected to a single lane (*L* = 1) with *N* = 1 to 10 motors as shown in Fig 5. An example of the simulation time course for *N* = 10 kinesin-1 motors on *L* = 1 microtubule lane is shown in Fig 5a. The nuclear extension, $dx_{\text{extension}}$, is calculated by the difference between the leading motors’ current and initial positions (dx(t=0)=0)) for Fig 5a. For Fig 5b, the extension is taken as that at the end of the simulation once the stall force has been reached and the motors have stopped moving. In Fig 5b we plot the mean final extension averaged over 100 simulation runs. This shows there is no extension (dx=0nm) for *N* = 1 to 3 and an extension of 8nm (one step) for *N* = 4 to *N* = 10. This suggests that a few motors on a single microtubule are too weak to deform the nucleus because the force, $f_{\text{spring}}=4.16pN$, generated by a single step of kinesin-1 (8nm) is larger than their stall force. A larger team of motors allows a step to be taken due to the crowding effect of following motors preventing the leading motor stepping backwards.

**Fig 6:**
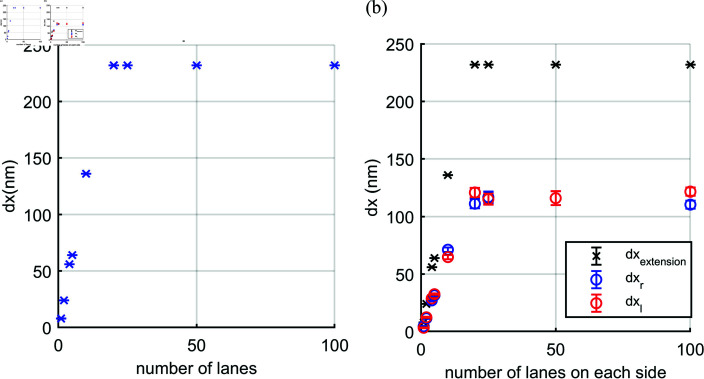
Nuclear extension (dx) when pulled (a) at one end with the other end fixed or (b) at both ends with increasing number of microtubules (lanes) from *L* = 1 to *L* = 100 and fixed total of 100 motors (*N*_*T*_ = 100) on each side, shared equally between the lanes on that side. The plot (b) shows the total nuclear extension $dx_{\text{extension}}$ is a sum of the nuclear extension by the right and left team *dx*_*r*_ and *dx*_*l*_, respectively.

We next show the effect of the number of lanes, *L*, on the nuclear extension when there are *N* motors on each lane. In Fig 6, we fix the total number of motors as *N*_*T*_ = 100 on each side and vary the number of lanes, L=1,2,4,5,10,20,25,50,100, with respectively N=100,50,25,20,10,5,4,2,1 motors each. When there is more than one lane (*L*>1), we assume the leading motors on each lane share force equally between them. Therefore, for multiple lanes the leading motors experience less force compared to the single lane case and can thus perform more steps and stretch the nucleus more. Fig 6a and 6b show the nuclear extension (*dx*) pulled by one team and two balanced teams, respectively. The results of nuclear extension pulled by one side and both sides are similar, but the extensions pulled by both sides of the nucleus is made up of the sum of the extension arising from the right and left sides ($dx_{\text{extension}}=dx_{r}$  +  *dx*_*l*_) as shown in Fig 6b. Both Fig 6a and 6b clearly show a larger extension with more lanes despite the total number of motors being fixed. This suggests that the nucleus can be stretched more if there are more microtubules even with the same total number of motors. However, a plateau is reached in Fig 6 when there are N≤5 motors on each lane. This implies that continuing to increase the number of lanes whilst keeping the total number of motors fixed (i.e. a decreasing number of motors per lane) cannot further increase the extension once the number of motors per lane becomes too small (N≤5 with our parameter values). Interestingly, Fig 6 suggests that 100 motors and ten microtubules could generate sufficient force to extend a nucleus by an amount visible with optical microscopy.

### 3.2. Nucleus pulled at both ends

If we consider a nucleus pulled in opposite directions by motors on its left and right sides this could result in extension and displacement. On each side of a simplified one dimensional nucleus it could be connected to a single or multiple microtubules by bound kinesin-1 motors. In the following, we consider a model nucleus pulled by balanced (Sect 3.2.1) and unbalanced (Sect 3.2.2) teams of motors at each end. The balanced teams are defined by having the same number of motors ($N_{r}=N_{l}$) and lanes ($L_{r}=L_{l}$) on each side. Unbalanced teams have differences in either motor number ($N_{r}\neq N_{l}$) or lane number ($L_{r}\neq L_{l}$) on the right versus the left side. The results of differing in motor number ($N_{r}\neq N_{l}$) are shown in S1 Text.

#### 3.2.1. Balanced pulling teams.

We simulate a model of the nucleus pulled by balanced teams at both ends ($N_{r}=N_{l}$, $L_{r}=L_{l}$). Fig 2 shows an example of a model nucleus pulling by balanced teams of four motors on a single lane on each side ($L_{r}=L_{l}=1$,$N_{r}=N_{l}=4$).

**Fig 7:**
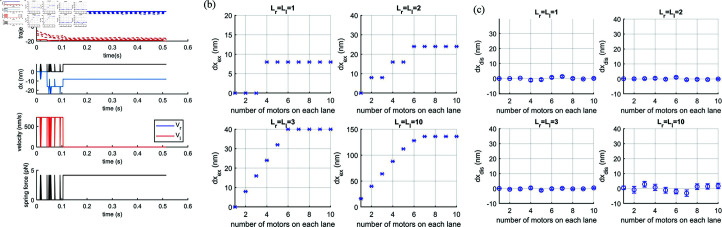
(a) (Top) An example of trajectories (lattice site numbers) for four motors ($N_{r}=N_{l}=4$) stepping along one lane on the left and one lane on the right ($L_{r}=L_{l}=1$). Blue and red solid lines represent the position in number of steps of the leading motor on the right and left lane, respectively. Blue and red dashed lines represent the position in number of steps of the following motors on the right and left lanes, respectively. (Second from the Top) The nucleus extension, $dx_{\text{extension}}$, (black) and displacement, $dx_{\text{displacement}}$, (blue) against time. Note that one step dx=8nm. (Third from the Top) velocity of the left (red) and right (blue) motor teams against time are exactly the same. (Bottom) Spring force against time (black). (b) Extension, $dx_{\text{extension}}$ (symbol x), and (c) displacement, $dx_{\text{displacement}}$ (symbol o), pulled by balanced teams against the number of motors on each lane for one, two, three and ten filaments (L=1,2, 3 and 10) on each side. The error bars are the standard error $\frac{\sigma }{\sqrt{n}}$ where σ is standard deviation and *n* = 100 is number of runs.

Trajectories of each motor on the right (blue) and left (red) are shown in Fig 7a for an example simulation run. On the same figure we also display the time course of nuclear extension and displacement, velocities of each motor team and the nuclear spring force. For this particular example, the initial and final position of the leading motor of right team (blue solid line) are at the same lattice site whereas those of left team (red solid line) move forward one step, i.e. *dx*_*r*_ = 0 and $dx_{l}=8nm$. This results in an extension $dx_{r}+dx_{l}=8nm$ and displacement $dx_{r}-dx_{l}=-8nm$ where the minus sign indicates the direction is towards the left. In Fig 7b and 7c we plot the mean extension and displacement calculated over 100 simulation runs against the number of motors on each lane for $L_{r}=L_{l}=\{1,2,3,10\}$ lanes. As expected for balanced teams, the translational displacement is zero on average. The extension appears to reach a plateau for *N*>5 for the parameters tested.

**Fig 8:**
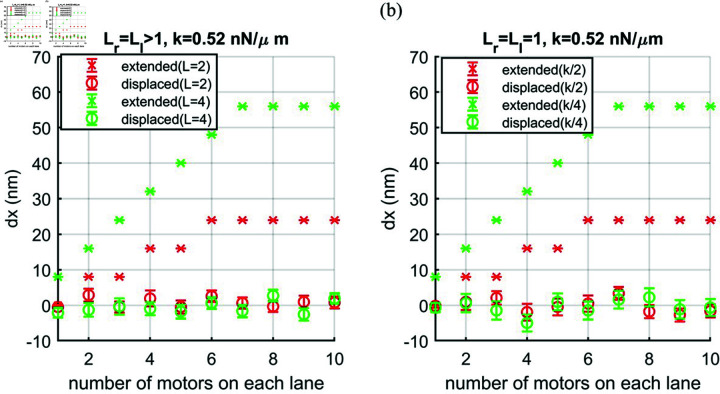
Nuclear extension (dx) pulled by two balanced teams on (a) multiple lanes ($L_{r}=L_{l}=2$ and $L_{r}=L_{l}=4$) with a constant nuclear spring constant *k* = 0.52 $nN\mu {\text{m}}^{-1}$ and (b) a single lane ($L_{r}=L_{l}=1$) with reducing nuclear spring constant (*k*/2 and *k*/4).

Moreover, it is interesting to compare the results of balanced pulling teams on multiple lanes to that on a single lane with reduced nuclear spring constant. From Fig 8a and 8b, it is clear that the results of a single lane $L_{r}=L_{l}=1$ with *k*/2 = 0.26 $nN\mu {\text{m}}^{-1}$ show the same as the results of multiple lanes $L_{r}=L_{l}=2$ with *k* = 0.52 $nN\mu {\text{m}}^{-1}$. Likewise, the results of a single lane $L_{r}=L_{l}=1$ with *k*/4 = 0.13 $nN\mu {\text{m}}^{-1}$ are the same the results of multiple lanes $L_{r}=L_{l}=4$ with *k* = 0.52 $nN\mu {\text{m}}^{-1}$. Our simulations show the expected trend of nuclear extension and displacement with lower spring constant resulting in smaller force as predicted by the theory, $f=1.87k\;dx_{\text{extended}}/L$.

#### 3.2.2. Unbalanced pulling teams.

**Fig 9:**
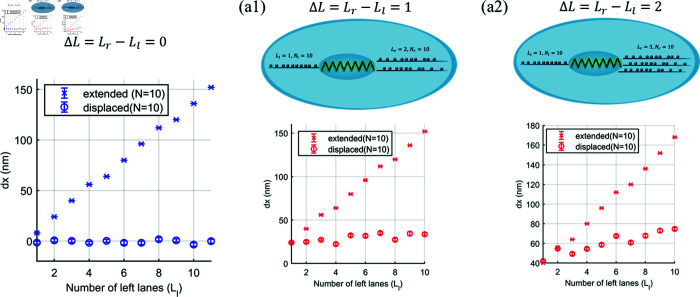
Nuclear extension and displacement pulled by different number of lanes between right and left ends with ten motors on each lane ($N_{r}=N_{l}=10$) is plotted against the number of left lanes (*L*_*l*_). The number of left lanes (*L*_*l*_) increases from 1 to 10, so the total number of motors on each side (*N*_*T*_) increases with *L*_*l*_. (a) Nuclear extension for balanced pulling teams ($L_{r}-L_{l}=0$) where $L_{r}=L_{l}=1$ to 11 clearly showing zero displacement as expected. (b) There is one more lanes on the right, $△L=L_{r}-L_{l}=1$, so the number of lanes on the right (*L*_*r*_) is from 2 to 11 in this case. (c) There are two more lanes on the right, $△L=L_{r}-L_{l}=2$, so the number of right lanes is from 3 to 12 in this case. This figure was created using icons from the Reactome Icon Library (https://reactome.org/icon-lib), available under a Creative Commons Attribution 4.0 International (CC BY 4.0) license.

In this section, we focus on cargo extension and displacement resulting from pulling by unbalanced teams at the right and left ends of the cargo. The unbalanced pulling team can differentiate in motor number and lane number. We demonstrate the effect of a difference in the number of motors ($N_{r}\neq N_{l}$) between a single right and left lane ($L_{r}=L_{l}=1$) and the effect of a difference in the number of lanes but the fixed total number of motors on the same side in S1 Text. From the simulation results in S1 Text, it obviously shows that the number of lanes has the strongest role in cargo extension and displacement. By placing N≥5 on each lane we can isolate the effect of the number of lanes. We then vary number of lanes and choose to have ten motors (*N* = 10) on each lane. In the study, we investigate one-lane ($△L=L_{r}-L_{l}=1$) and two-lane (△L=2) differences between right and left sides for *L*_*l*_ = 1 to 10, compared with the zero-lane differences (△L=0), see Fig 9. The results show that the extension and displacement increases linearly with the increasing number of lanes (and number motors since there are ten on each lane) in both cases. We can see that the nuclear extension of the unbalanced team in Fig 9b shows the same amount of nuclear extension (dx) as that of the balanced teams of the side having more lane numbers if the number of motors is large enough. This is because the more lanes each side has, the smaller the force shared among the leading motors. This allows more nuclear extension until the sharing force is larger than the stall force of a collective motor in each lane.

As expected from Fig 9b and 9c, the nuclear displacement is greater for the case of △L=2 than the case of △L=1 due to the force being shared between more lanes and the zero displacement of the balanced pulling team. As we increase the number of lanes on each side of the nucleus then the extension increases and the displacement increases if the number of lanes is different on each side i.e. the more microtubules on each side the larger the extension and the greater the difference in number of microtubules on each side the larger the displacement. This is likely to be relevant in cells as we would expect more microtubules on the side where the centrosome is located.

### 3.3. Application to *in cellulo* experiments

From our simulation results, we can see that a larger nuclear extension can be caused by either an increase in the number of microtubules or an increase in the number of active kinesin-1 motor proteins. In order to test whether something similar happens *in cellulo*, we treated cells with the drug kinesore at a concentration of 50 μM. It is known that kinesore activates the molecular motor kinesin-1 and its function of controlling microtubule dynamics in cells [57, 58]. Andreu-Carbó, *et al*. showed that adding kinesore to cells increased the binding of kinesin-1 to microtubules and the movement of kinesin-1 along microtubules [58]. This also caused an increase in the exchange of tubulin dimers along the microtubule shaft inducing rescue events [58]. A rescue is when a depolymerizing microtubule starts to polymerize again. As a result of an increased number of rescue events, microtubules are longer and have an increased lifetime in cells treated by kinesore and Andreu-Carbó, *et al*. [58] also show that this results in an increase in the density of the microtubule network. In our model we assume microtubule length and simulation time is unlimited. During the course of a single simulation we do not change the number of microtubules but we vary this between simulations. We mimic the effect of kinesore by an increase in the number of kinesin-1 and microtubules in our simulations. In our model this results in nuclear deformation. We therefore analyzed images of cells with and without the kinesore drug treatment to measure the length of the fluorescent nucleus in each case.

**Fig 10:**
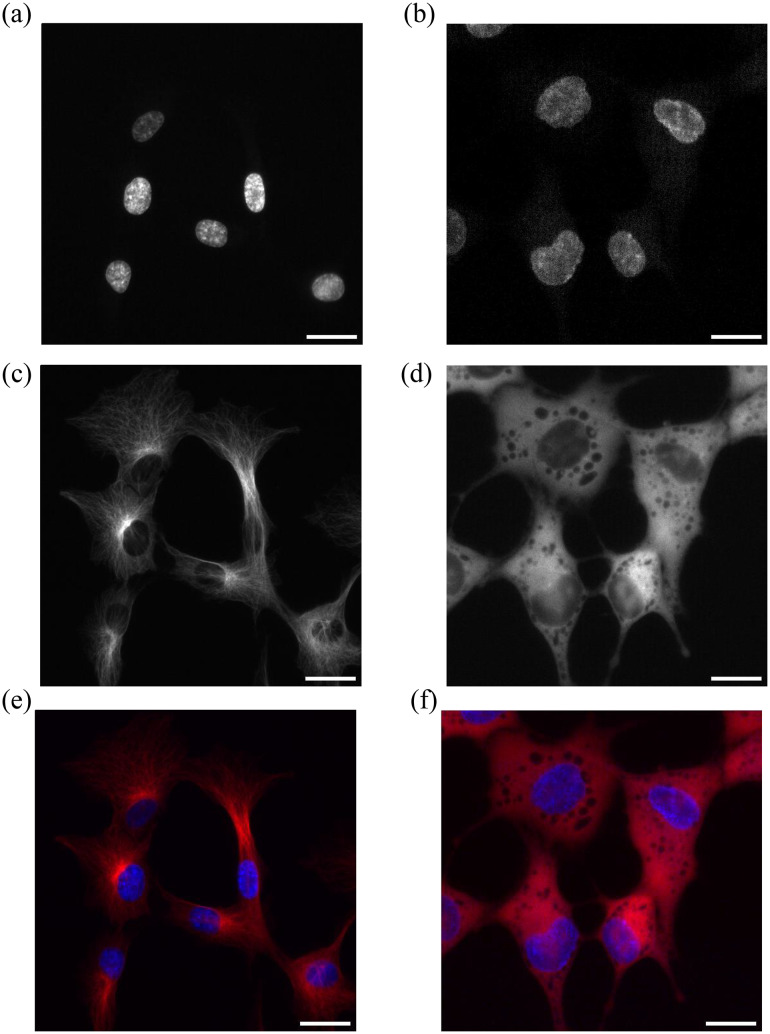
(a) (Left) Nuclei of cells in control conditions (b) (Right) Nuclei of cells with kinesore drug treatment at 50 μM. (c) (Left) Microtubules of cells in control conditions (d) (Right) Microtubules of cells with kinesore drug treatment at 50 μM. The nucleus is blue and microtubules are red (e) (Left) in control condition and (f) (Right) in kinesore drug treatment. Scale bars are all 25 μm. More details in Sect 2.4.

**Fig 11:**
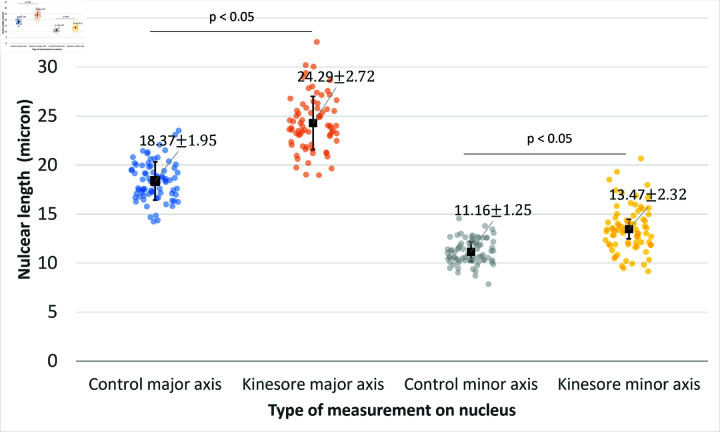
Experimental results of nuclear length along major and minor axis comparing between cell with and without kinesore treatment.

An example of images of nuclei (blue) in control cells and cells with kinesore treatment are shown in Fig 10a and 10b, respectively. From the experimental images, it can be seen that the nucleus of cells with kinesore treatment are larger than that without kinesore treatment. We measured the nuclear length along major and minor axis using image J. We calculated the mean average nuclear extension of 74 examples of cells with kinesore treatment compared to cell without kinesore treatment (control case). We found that cells with kinesore drug treatment have nuclei that are significantly longer in both the major and minor axis. The results show that nuclei in cells with kinesore treatment are extended by 5.9μm and 2.4μm in the major (p-value = $4.92\times 10^{-32}$) and minor axis (p-value = $4.86\times 10^{-12}$), respectively, compared to the control cases as shown in Fig 11.

**Fig 12:**
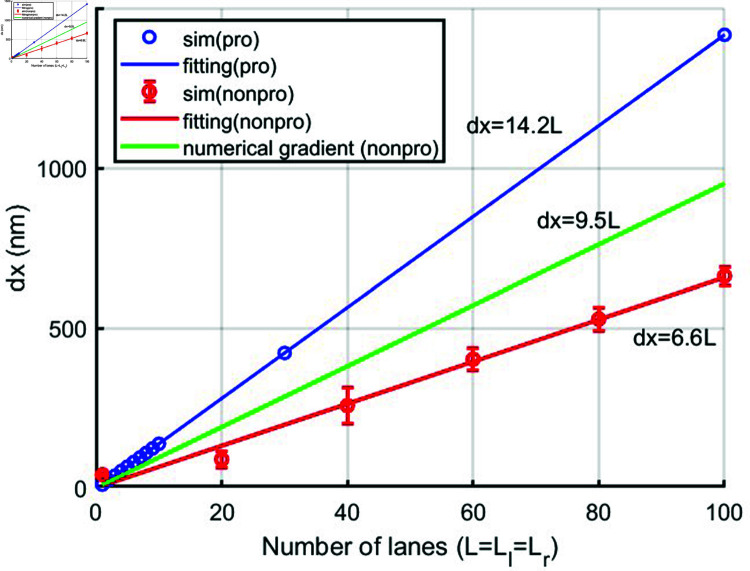
Nuclear extension (dx) against number of microtubules on the left and right (lanes, $L=L_{l}=L_{r}$) pulled by balanced teams, for which *L*_*l*_=*L*_*r*_, with ten motors on each individual microtubule. The blue circles are simulations for perfectly processive motors and the blue line is the theory. The red circles are simulations for partially processive motors and the red line is a fitting line and the green line is the theory for partially processive motors.

Fig 10c and 10d shows microtubules (red) in cells without and with kinesore treatment respectively. The microtubule network in cells treated by kinesore appears different from that in control cells, although it is difficult to specify microtubule number between control cells and kinesore treated cells from the experimental images. Hence, we used our simulation to estimate the number of microtubules resulting in the observed nuclear extension on the major and minor axis. We assume that the nucleus is extended but there is no translocation. We therefore assume it is extended by balanced pulling teams which have the same number of motors and microtubules on each side of the nucleus. In order to reduce the number of factors involved, we eliminated the effect of the number of motors per filament. We achieved this by assuming that there are enough motors on each microtubule. From our results, we know that if the number of motors on each individual microtubule is large enough then further increasing the number of motors per filament does not increase the nuclear extension. We therefore specify ten motors on each individual microtubule. We simulate both perfectly processive and partially processive kinesin-1s. We calculate the nuclear extension, dx, for an increasing number of microtubules (lanes, L) and motors (ten motors per lane) on each side of the nucleus, see Fig 12.

For perfectly processive kinesin-1s, we find the nuclear extension, $dx_{\text{extension}}$, increases linearly with the number of lanes on each side, *L* (Fig 12). Our simulation points lie on the line $dx_{\text{extension}}=14.2L$ which we calculate from the theory. By setting the velocity $V_{N}=0$ to zero in Eq 1 we find the stall force $f_{\text{stall}}=N\ln\frac{p}{q}=\frac{1.87\times f_{\text{spring}}}{L}=\frac{1.87kdx_{\text{extended}}}{L}$ where we have set the spring force $f_{\text{spring}}=kdx_{\text{extended}}$ and 1.87 is the nondimensionalization factor. This gives us the gradient =$\frac{f_{\text{stall}}}{1.87k}=23.0$ since the stall force for 10 motors is 12.2pN and k=0.52nN/μm. However, in our simulations we used a threshold for which the motors stop moving, not zero as used in the rough calculation above. We show the full calculation using the threshold of $|\frac{V_{f_{s}}}{V_{f_{0}}}|\leq 10^{-5}$ to determine the simulation stall force in S1 Text which gives the gradient =14.2 as in Fig 12. Using this linear equation for the nuclear extension we predict the number of microtubules needed to result in the observed nuclear extension of 5.9μm is 415 more microtubules on each side of the nucleus along the major axis of nucleus and 2.4μm is 169 more microtubules on each side along the minor axis of nucleus. It is important to note that, due to the assumptions involved in the simple model, these predicted numbers should not be interpreted as an accurate prediction of the number of microtubules. Rather, we give these numbers to provide an idea of the maximum number of microtubles predicted by the model and offers a qualitative understanding of how motor forces contribute to nuclear deformation. These numbers are reasonable given that the number of kinesins motors per neuron is estimated to be 10^5^ to 10^7^ [3]. The main limitation of our model is that it is a one dimensional model of a three dimensional system. As such the model assumes the effect of microtubules in all directions can be projected onto the long and short axes with appropriate geometric factors. This will affect the quantitative predictions but not the qualitative predictions. However, for a better treatment a higher-dimensional model should be developed. This would need to model the mechanical properties of nucleus in 3D and and the 3D arrangement of microtubules, which is beyond the scope of the current study.

To include the biochemistry of partially processive kinesin-1s, we consider the binding and unbinding behaviors of molecular motors on microtubule filaments. The binding and unbinding rates of the kinesin-1 motors are 5 $s^{-1}$ [4, 5] and 1 $s^{-1}$ [4], respectively. We give further validation details for our simulation of partially processive motors for binding/unbinding and stepping behaviors in S2 Text. We find that the nuclear extension $dx_{\text{extension}}$ linearly increases with the number of lanes on each side, *L*, just like for perfectly processive motors (Fig 12). The line of best fit to our simulation results is $dx_{\text{extension}}=6.6L$. To compare to our analytical theory we set Eq 2 with maximum *N* = 10 partially processive motors to the velocity threshold we used in the simulations and substitute *M* = 8.6 as the average number of binding sites per lane. The numerical solution gives the stall force = 9.3 corresponding to the numerical gradient shown in Fig 12 for partially processive kinesin-1 motors as $\text{gradient}=\frac{\text{stall force}}{1.87k}=9.5$, which is larger than the gradient of the fit to the simulation points. There are two reasons why our simulation results have a lower gradient than the numerical solution to our analytical equation. One important reason is that with only a few motors bound, a single step by kinesin-1 can exceed its stall force [18] causing the simulation to stop before more motors are bound. However, if a sufficiently large number of partially processive motors are bound, they will become densely packed (crowding), making it difficult for the leading motor to step backwards. Partially processive motors detach leading to a weaker crowding effect compared to perfectly processive motors. This leads to a greater extension for perfective processive motors compared to partially processive motors. This biases the distribution of bound motors to small numbers of bound motors, as seen in the plot of *P*(*n*) for *L* = 1 in S2 Text. This causes fewer bound motors than the analytical expression for *P*(*n*). Another difference is caused by a variable *M* binding sites. In the analytical expression *M* binding sites is substituted by its average. However, in the simulations the available number of binding sites *M* changes every step. To preserve the sequence during stepping on lanes, a motor can only bind to unoccupied sites between its neighboring motors. The number of accessible binding sites changes over time following the first and last motors’ positions. Additionally, we allow one more site in front/behind the position of the fourth/back following motors. However, only one more site in front of the occupied motors is allowed for the leading motor. The sequence preservation leads to fewer available binding sites than that given by the average *M* in the analytical expression, shifting the distribution *P*(*n*) to lower *n*. This reason was discussed in [18] to explain why the simulation *P*(*n*) is less than the analytical *P*(*n*). Overall this results in a lower gradient in Fig 12 for the simulation results compared to the numerically solved analytical equation. Using the linear relationship between nuclear extension and number of lanes for simulated partially processive kinesin-1s, the observed nuclear extension requires approximately twice (14.2/6.6) as many microtubules compared to that caused by perfectly processive kinesin-1s. Note that in this comparison we are using a maximum number of bound motors *N* = 10 per lane for partially processive motors compared to *N* = 10 motors per lane actually bound for perfectly processive motors, i.e. the total number of motors per lane is the same in each case.

## 4. Conclusion

We present a model to study the effect of force generated by molecular motors stepping along cytoskeletal filaments on cargo displacement and extension. We apply our model to investigate whether molecular motors that transport cargo are strong enough to move and deform the largest cellular cargo, the nucleus. We use the tug-of-war paradigm describing the mechanical competition between two opposing teams of molecular motors. However, instead of considering two different molecular motor species moving in different directions, we focus on a single type of motor on oppositely directed microtubules. We consider microtubules attached to left and right sides of the nucleus in opposite directions with molecular motors attached to the nucleus generating pulling forces along the microtubules. We use a Monte Carlo simulation with a Gillespie algorithm to simulate a simple exclusion process of motors on a one dimensional lattice along a microtubule. We verify that cargo pulled by teams consisting of the same number of motors and microtubules at both ends are deformed but not displaced and that unbalanced teams can lead to net displacement and extension. Our results show that motors on a single microtubule are not strong enough to change the nucleus’s shape and position, but that by increasing the number of microtubules on which molecular motors step displacement and extension can be achieved. Nuclear translocation can be induced by unbalanced teams of motors with more microtubules on one side than the other. Our model results corresponded well with experimental results showing that nuclei of cells treated by kinesore resulting in more kinesin-1 bound on more microtubules are extended in 2D compared with the control case (no kinesore treatment). We present results for both perfectly processive (absence of motor detachment) and partially processive (with binding and unbinding) motors. The extension of the nucleus follows the same trend in both cases, i.e. a linear increase in extension with number of microtubules. However partially processive motors need around twice as many lanes to achieve the same extension as perfectly processive motors assuming the same number of total motors per lane.

## Supporting information

S1 Text Perfectly processive motors. A detailed explanation of the behavior of perfectly processive motors including the effect of differences in the number of motors (Fig A in S1 Text) and lanes (Fig B in S1 Text) of the unbalanced pulling team, and the derivation of the gradient in Fig 12 with zero velocity and with the velocity threshold of balanced pulling team.
(PDF)

S2 Text Partially processive motors. Analysis of partially processive motor behavior, including binding probability distributions and comparison with processive motors. Results shown in Fig A in S2 Text.
(PDF)
