# Supplementary material for: Nuclear deformation by microtubule molecular motors
Source: PLoS Comput Biol. 2025 May 8;21(5):e1012305. doi: 10.1371/journal.pcbi.1012305 (PMC12101784; doi:10.1371/journal.pcbi.1012305)
Supplement: S1 Text — A detailed explanation of the behavior of perfectly processive motors including the effect of differences in the number of motors (Fig A in S1 Text) and lanes (Fig B in S1 Text) of the unbalanced pulling team, and the derivation of the gradient in Fig 12 with zero velocity and with the velocity threshold of balanced pulling team. (PDF) [file pcbi.1012305.s001.pdf]

Nuclear deformation by microtubule molecular motors:
Supporting Information
S1 Text: Perfectly processive motors

Unbalanced pulling team

The effect of different number of motors

Examples of cargo pulled by different numbers of motors on a single left and right lane are illustrated in Fig A (a). We place the stronger team on the right, so $N_r > N_l$. We run simulations with fixed N_l and increasing N_r such that the difference between motor numbers ($dN = N_r - N_l$) increases from 1 to 10. The results for nuclear extension and displacement pulled by a small left team and larger right motor team are shown in Fig A (b) and (c), respectively. The results show that a nucleus pulled by teams differing in the number of motors between each end show some extension and non-zero mean displacement. However, interestingly, if the number of motors on the each side is at least five ($N_l \geq 5$) then they act like a balanced team resulting in zero mean displacement, even though the right team is larger (Fig A (c)). Furthermore, increasing the number of motors on each side beyond five does not increase the extension (Fig A (b)). This corresponds to the results in [1] in which the velocity of processive motors when ($N_l \geq 5$) is indistinguishable.

The effect of different number of lanes

We simulate the model of a nucleus pulled by two opposite teams with a different number of microtubules (lanes) on each side, but with an equal total number of motors, N_T, on each side, as illustrated in Fig B (a). We show two cases of $N_T = 12$ motors on each side. The first case (blue) is $L_l = 2, N_l = 6$ and $L_r = 3, N_r = 4$ and the second case (orange) is $L_l = 3, N_l = 4$ and $L_r = 4, N_r = 3$. Both cases show the same extension but different displacements (means averaged over 100 runs), shown in Fig B (b). The first case shows more displacement than the second case because the ratio of shared force between right and left teams of the first case is larger than that of the second case.

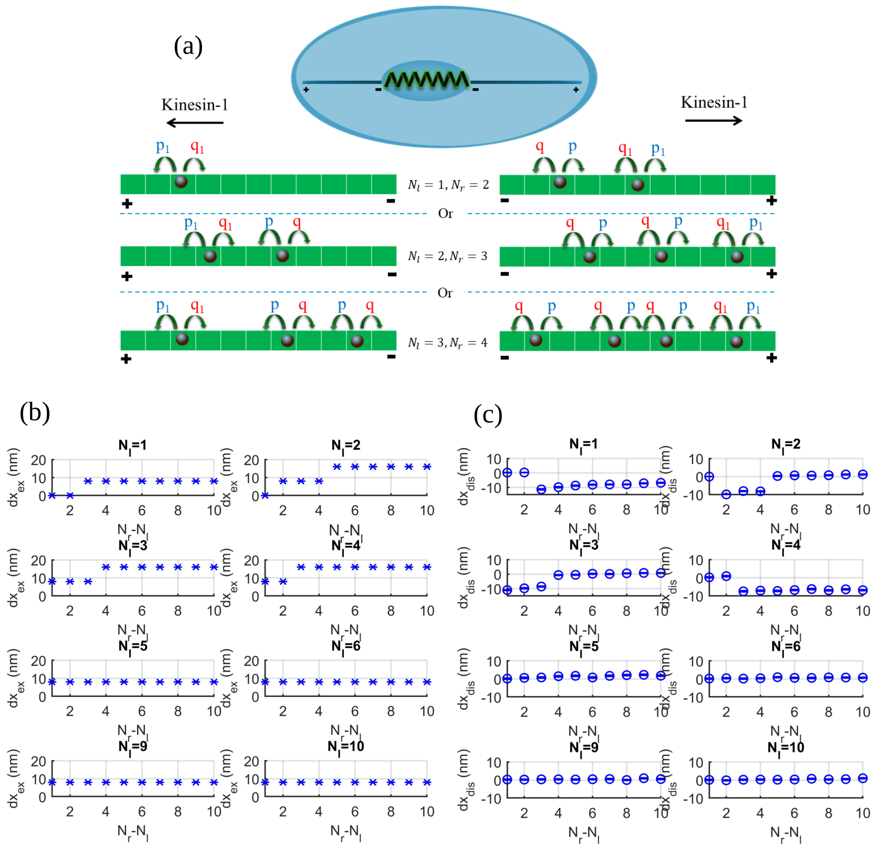

Figure A: (a) Diagram showing the nucleus pulled between a smaller left team and larger right team on one lane ($L_r = L_l = 1, N_r > N_l$). (b) The extension and (c) displacement of the nucleus pulled by unbalanced teams with varying $dN = N_r - N_l$ from 1 to 10. All points are means of 100 simulation runs. This figure was created using icons from the Reactome Icon Library (https://reactome.org/icon-lib), available under a Creative Commons Attribution 4.0 International (CC BY 4.0) license.

Balanced pulling team

The gradient in Fig.12 with zero velocity

When a cluster of N processive motors on L microtubules pulling each side of nucleus stop moving, it means $V_N = 0$. We obtain analytically the force at which motors stop moving called the stall force, f_{stall}, by setting $V_N = 0$ as follows,

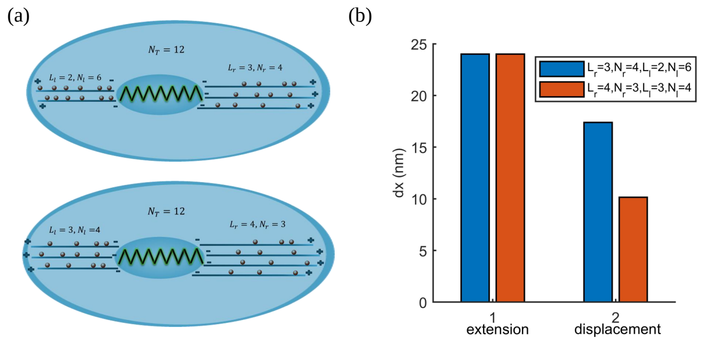

Figure B: (a) Diagram showing a nucleus pulled by unbalanced teams in which the total number of motors on each side is equal ($N_T = 12$) but the number of lanes on each side is different ($L_l \neq L_r$). The first case (blue) is $N_r = 4, L_r = 3, N_l = 6, L_l = 2$ and the second case (red) is $N_r = 3, L_r = 4, N_l = 4, L_l = 3$. (b) The bar graph shows the extension and displacement of the nucleus for these two cases. This figure was created using icons from the Reactome Icon Library (https://reactome.org/icon-lib), available under a Creative Commons Attribution 4.0 International (CC BY 4.0) license.

$$
\begin{aligned}
V_N &= p\frac{(1-e^{f}(q/p)^N)(1-q/p)}{e^{f\delta}(1-q/p)+e^{f}(q/p-(q/p)^N)} \\
0 &= 1-e^{f_{\text{stall}}}(q/p)^N
\end{aligned}
$$

and therefore

$$
f_{\text{stall}} = N\ln\frac{p}{q} = \frac{1.87\times f_{\text{spring}}}{L} = \frac{1.87k\,dx_{\text{extended}}}{L} \tag{1}
$$

where 1.87 is a factor converting the spring force into a dimensionless unit of motor force. If we plot a graph of nuclear extension, dx_{extended}, against number of microtubules, L, (as in Fig. 12) then the gradient $=\frac{f_{\text{stall}}}{1.87k}$. Therefore, we can find the gradient analytically as;

$$
\text{gradient} = \frac{f_{\text{stall}}}{1.87k} = \frac{N\ln\frac{p}{q}}{1.87k} = \frac{10\ln\frac{100}{10}}{1.87\times 0.52} = 23.03 \approx 23. \tag{2}
$$

However, in our simulation, we take the stall force to be when the velocity reaches a given threshold (not exactly zero as in the solution above). The simulation result in Fig. 12, shows that the gradient is not close to 23 so we need to calculate it accounting for the threshold velocity.

The gradient in Fig. 12 with the velocity threshold

In our simulation, we take the stall force to be when the velocity reaches a given threshold instead of exactly zero. The stall force is affected by this threshold. For kinesin-1, its forward and backward stepping rates are $p = 100\text{s}^{-1}$, $q = 10\text{s}^{-1}$, respectively. It is therefore reasonable to assume that $p \gg q$ and so $(\frac{q}{p})^N$ in the case of $N = 10$ can be neglected compared to q/p in the velocity which can be written as follows,

$$V_N = p\frac{(1 - e^f(q/p)^N)(1 - q/p)}{e^{f\delta}(1 - q/p) + e^f(q/p - (q/p)^N)}$$

$$V = \frac{p(1 - \frac{q}{p})}{e^{f\delta}(1 - \frac{q}{p}) + e^f(\frac{q}{p})} \text{ where } d = 0.5$$

$$e^{\frac{f}{2}}(1 - \frac{q}{p}) + e^f(\frac{q}{p}) = \frac{p(1 - \frac{q}{p})}{V}$$

$$x(1 - \frac{q}{p}) + x^2(\frac{q}{p}) - \frac{p(1 - \frac{q}{p})}{V} = 0 \text{ where } x = e^{\frac{f}{2}}$$

$$x = e^{\frac{f}{2}} = \frac{p}{2q}[-(1 - \frac{q}{p} + \sqrt{(1 - \frac{q}{p})^2 + 4(\frac{q}{p})\frac{p(1 - \frac{q}{p})}{V}}]$$

$$f = 2\ln\frac{p}{2q}[(\frac{q}{p} - 1) + \sqrt{(1 - \frac{q}{p})^2 + \frac{4q(1 - \frac{q}{p})}{V}}]$$

$$f = 2\ln\frac{100}{20}[(\frac{10}{100} - 1) + \sqrt{(1 - \frac{10}{100})^2 + \frac{4(1 - \frac{10}{100})}{V}}]$$

In the simulation, the threshold is set up as 10^{-5}, $\frac{V}{V_o} = 10^{-5}$, where V is the velocity of a group of motor at each time step and V_o is the velocity at initial time. At initial time, $f = 0$, $V_o = 90$, so the velocity at final time step is $V = 9 \times 10^{-4}$. Therefore, we obtain

$$\text{gradient} = \frac{f_{\text{stall}}}{1.87k} = \frac{2\ln 5[-0.9 + \sqrt{(0.9)^2 + \frac{4\times10\times0.9}{9\times10^{-4}}}]}{1.87 \times 0.52} = 14.2 \qquad (3)$$

This is the gradient after we apply the threshold for calculating velocity of motors for each time steps in the simulations. Note that this is the value of the gradient in Fig.12.

References

[1] Rueangkham N, Estabrook ID, Hawkins RJ. Modelling cytoskeletal transport by clusters of non-processive molecular motors with limited binding sites. R Soc Open Sci. 2020;7(8):200527. doi:10.1098/rsos.200527 .

.
