# Supplementary material for: Nuclear deformation by microtubule molecular motors
Source: PLoS Comput Biol. 2025 May 8;21(5):e1012305. doi: 10.1371/journal.pcbi.1012305 (PMC12101784; doi:10.1371/journal.pcbi.1012305)
Supplement: S2 Text — Analysis of partially processive motor behavior, including binding probability distributions and comparison with processive motors. Results shown in Fig A in S2 Text. (PDF) [file pcbi.1012305.s002.pdf]

Nuclear deformation by microtubule molecular motors: Supporting Information S2 Text: Partially processive motors

We use the distribution shown in [1] for n out of a maximum of N nonprocessive motors binding on microtubules within a limited number of binding sites (M);

$$P_n(N,M) = \frac{N!M!}{n!(N-n)!(M-n)!}\left(\frac{k^s_{\text{on}}}{k_{\text{off}}}\right)^n P_0 \tag{1}$$

where P_0 is the normalization constant given by $\sum_{n=0}^{N} P_n(N,M) = 1$. For $M >> n$, Eq. 1 becomes the binomial distribution for an unlimited number of binding sites.

We plot the probability distribution of bound motors, $P_n(N,M)$ for $N = 10$ and $L = 1$ in Fig A (a) in which a spring force of $f_{spring} = k(dx_r + dx_l)$ is changed following motors' stepping, right (dx_r) and left (dx_l) teams. The parameter values of the kinesin-1 motors are taken as $k_{on} = 5\text{s}^{-1}$, $k_{off} = 1\text{s}^{-1}$, $p = 100\text{s}^{-1}, q = 10\text{s}^{-1}$ and $\delta = 0.5$. We can see that $P(n)$ is biased towards a few bound motors. This likely due to the simulation stopping quickly, before having as many bound motors as predicted by the analytical binomial distribution with limited binding sites (M) given in Eq. 1.

For comparison, we plot the probability distribution of the bound motors $P_n(N,M)$ for $N = 10$ at a zero spring force ($f = 0$) in Fig A (b) and (c) for $L = 1$ and $L = 5$, respectively. The parameter values of the kinesin-1 motors are $k_{on} = 5\text{s}^{-1}$, $k_{off} = 1\text{s}^{-1}$, $p = 100\text{s}^{-1}, q = 10\text{s}^{-1}$ and $\delta = 0.5$. In the simulation, the motors are allowed to swap positions during re-binding but preserve the sequence during stepping. The M binding sites are changed following the bound/moved leading and last motor positions and substitute on average in the analytic $P_n(N,M)$. Fig A (b) and (c) show consistency between the simulation P_n and the analytical P_n because there is no an effect of forces that cause the simulation to stop early.

We validated the simulation of patially processive motors with a very small unbinding rate by using parameter values of $p = 100\text{s}^{-1}, q = 10\text{s}^{-1}, k_{on} = 1000\text{s}^{-1}$ and $k_{off} = 0.1\text{s}^{-1}$ and measure its nuclear extension (dx) as shown in Fig A (d). We found that the nuclear extension (dx) of the simulation of the partially processive motor with an unbinding rate of $k_{off} = 0.1\text{s}^{-1}$ is indistinguishable from that of the simulation of processive motors. Thus, partially processive motors with very small unbind rates behave like processive motors, as expected. This provides a validation of the non-overlapping stepping behavior of molecular motors following a simple exclusion process in our simulation

model.

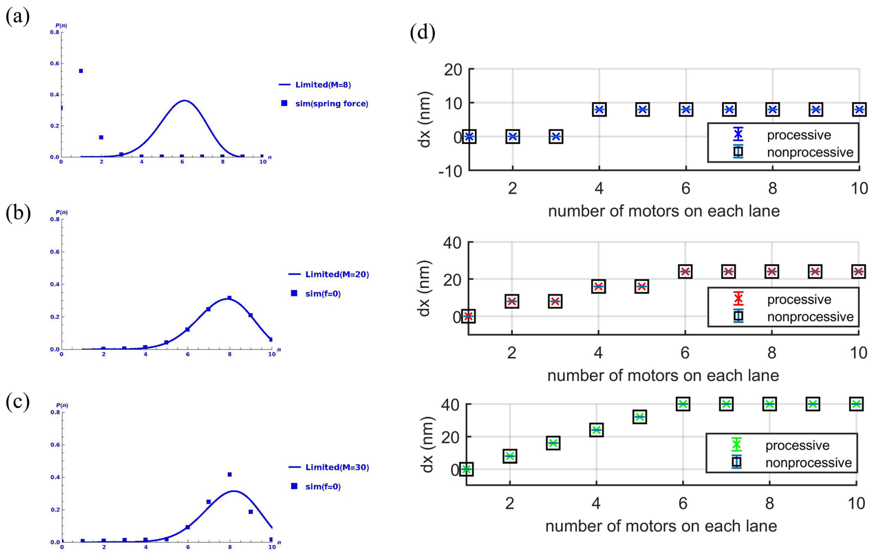

Figure A: (a) Probability distribution of partially processive motor simulation with spring force $f_{spring} = k(dx_r + dx_l)$ changed by motors' steps of right dx_r and left team dx_l and variable number of binding sites, $M(t)$, from simulation results (square) comparing with binomial distribution with limited binding sites averaged over the 100 simulation runs (blue line) for a single lane ($L = 1$). (b-c) Probability distribution of partially processive motor simulation with no exerted force $f = 0$ and variable number of binding sites, $M(t)$, from simulation results (square) comparing with binomial distribution with limited binding sites averaged over the 100 simulation runs (blue line) for (b) $L = 1$ and (c) $L = 5$. The parameters for (a-c) are $p = 100\text{s}^{-1}, q = 10\text{s}^{-1}, k_{on} = 5\text{s}^{-1}$ and $k_{off} = 1\text{s}^{-1}$. (d) Extension, $dx_{\text{extension}}$ (symbol □) pulled by a group of N nonprocessive kinesin motors on each lane for one, two and three filaments ($L = 1, 2$ and 3) on each side of the nucleus. It is compared to the extension, $dx_{\text{extension}}$ (symbol x) of a group of N processive motors on each lane for one, two and three filaments ($L = 1, 2$ and 3) on each side of the nucleus. The parameters are $p = 100\text{s}^{-1}, q = 10\text{s}^{-1}, k_{on} = 1000\text{s}^{-1}$ and $k_{off} = 0.1\text{s}^{-1}$. The standard error is also plotted.

References

[1] Rueangkham N, Estabrook ID, Hawkins RJ. Modelling cytoskeletal transport by clusters of non-processive molecular motors with limited binding sites. R Soc Open Sci. 2020;7(8):200527. doi:10.1098/rsos.200527 .
